# Comparison of Self-Reported Sexual Activity Among Heterosexuals with Sexual Spread of Poorly Transmittable Agents: A Minimalistic Approach to Estimating Sexual Activity Based on HIV Incidence

**DOI:** 10.3390/ijerph17155504

**Published:** 2020-07-30

**Authors:** Andreas Hahn, Christoph Kröger, Christian G. Meyer, Ulrike Loderstädt, Thomas Meyer, Hagen Frickmann, Andreas Erich Zautner

**Affiliations:** 1 Institute for Medical Microbiology, Virology and Hygiene, University Medicine Rostock, 18057 Rostock, Germany; hahn.andreas@me.com (A.H.); frickmann@bnitm.de (H.F.); 2 Department of Clinical Psychology and Psychotherapy, University of Hildesheim, 31141 Hildesheim, Germany; christoph.kroeger@uni-hildesheim.de; 3 Faculty of Medicine, Duy Tan University, ĐàNẵng 550000, Vietnam; christian.g.meyer@gmail.com; 4 Institute of Tropical Medicine, Eberhard Karls University, 72074 Tübingen, Germany; 5 Vietnamese-German Center of Medical Research, Hanoi 113601, Vietnam; 6 Diagnostic Department, Bernhard Nocht Institute for Tropical Medicine Hamburg, 20359 Hamburg, Germany; ulrike.loderstaedt@bnitm.de; 7 Clinic of Dermatology, St. Josef-Hospital, Ruhr-University, 44791 Bochum, Germany; t.meyer@klinikum-bochum.de; 8 Department of Microbiology and Hygiene, Bundeswehr Hospital Hamburg, 22049 Hamburg, Germany; 9 Institute for Medical Microbiology, University Medical Center Göttingen, 37075 Göttingen, Germany

**Keywords:** modeling, sexual transmitted infection, HIV, sexual activity, transmission

## Abstract

The aim of this study was to assess whether epidemics of sexually transmitted infections caused by poorly transmittable agents corresponded to self-reported sexual activity in a distinct population. To exemplify this, a model was used to investigate whether HIV infection incidences corresponded to the extent of sexual activity as assessed by a questionnaire-based study. The model suggested between 97 and 486 sexual contacts per German individual during a sexually active lifetime based on the annual HIV incidence of 680 among the heterosexual population reported by the German National Health Authority. This is in line with the estimated 296 sexual contacts during one’s lifetime, which was indicated by questionnaire respondents. The model confirms the correspondence of self-reported sexual activity with HIV incidence as reported by the German National Health Authority. Accordingly, HIV incidence- and prevalence-based modeling of sexual activity in a population provides crude estimations in situations where a range of uncertainty is acceptable. The model’s veracity is limited by a number of assumptions necessitated by the paucity of data. Nevertheless, the model may be suitable in settings where severe reporting bias has to be expected for legal or socio-cultural reasons.

## 1. Introduction

The extent of risky sexual activity that causes the spread of sexually transmitted infections (STIs) [1] is of significance for the design and implementation of preventive strategies. In order to adequately estimate transmission risks in distinct populations, it is important to quantify the extent of sexual activity and the associated increased risk of acquiring and transmitting STIs.

Several interview- or questionnaire-based studies on human sexual behavior, mostly from Western societies, have been published in recent years [2,3,4,5]. Such reports provide relevant insights into the sexual behavior of the populations studied. Although fidelity is an important value for the majority of participants, they also reported extradyadic sexual contacts, implying the possibility of acquiring STIs [6,7,8,9,10,11]. In Germany, 15–26% of women and 17–32% of men of reproductive age (16–47 years) within a representative cohort reported extradyadic sexual contacts in their current relationship [12]. These rates are slightly higher than those reported in the USA, but similar to those reported in other European countries [12].

Sources of reporting bias such as social acceptability can be identified but are difficult to control in such studies. A recent study on sexual behavior in Germany defined a standard population of Germans with an average of 10.2 female sexual partners per male and 5.5 male sexual partners per female study participant, but also a population at high-risk for STI acquisition with 38.4 female sexual partners per male and 17.3 male sexual partners per female respondent [2]. As indicated by social network analysis [1], it is a self-evident fact that a higher number of sexual partners is associated with a higher risk of contracting a STI. Reporting bias is suggested simply by looking at the proportions. There is an obvious similarity of the numbers of partners for men in the “standard population” (number of female sexual partners *n* = 10.2), which represented the vast majority of male respondents, and the female high-risk population, which represented a minority of female respondents with considerably more male sexual partners (*n* = 17.3) than the average female. When summing up male-to-female sexual contacts and female-to-male sexual contacts in defined populations, the sums should be similar. Considering the reports, however, such a similarity would require a large female high-risk population in contrast to a rather small male high-risk population. While this is theoretically possible, it conflicts with 21% of interviewed males, but only 15% of females reporting sexual contacts outside relationships in the same study [2]. Recently published reports on sexual activity in Germany between 2005 and 2016 suggest declining sexual interest in both women [13] and men [14], while newer data on the German population are still insufficient to allow conclusive analyses [15]. Overreporting from males and/or underreporting from females could be alternative explanations. Reporting bias is an issue in questionnaire-based assessments of sexual behavior and different methods of data acquisition may lead to different results. Current research addresses this problem in order to allow more representative, realistic, and comparable estimations in future studies [3].

The problem of reporting bias in questionnaire-based studies on sexual behavior can affect the reliability of such approaches [16]. Because of this, it is a challenge to carry out interview- or questionnaire-based studies of sexual behavior in all socio-cultural contexts [17]. For example, it may be difficult to obtain honest answers regarding extramarital sexual affairs or same-sex sexual contacts in societies where such behavior is legally proscribed and possibly threatened with harsh judicial sentences. In many tropical settings, taboos in societies with marked social or religious disapproval of such behavior may also play a role [18]. Accordingly, reliance solely on bias-prone self-reporting may lead to biased assumptions, which could negatively affect decisions concerning preventive medical programs. Although techniques to improve the reliability of such approaches have been suggested for some time [19], detailed discussion of these would be beyond the scope of this study. Consequently, methods less dependent on individual interviewees’ responses are desirable in identifying populations at increased risk of acquiring STIs.

For an interviewee-independent approach based on poorly transmittable STIs (a model for which is exemplified below), precise knowledge is necessary for population-specific prevalence and incidences regarding a particular STI, its transmission dynamics, the proportion of therapeutically cleared or suppressed infections with a reduced transmission risk, and the proportion of transmissions according to the sexual route. These variables should be considered in modeling and extracted from the literature. One option is to retrieve incidences and prevalence of STIs from national health agency databases, although publications on the up to date STI-epidemiology are also available [20]. Transmission dynamics (i.e., likelihood of transmission to occur depending on other variables, such as disease activity, pathogen load, and specific mode of sexual contact) have to be retrieved from the literature for each assessed STI [1].

Although STI transmission via non-sexual routes can theoretically occur, if an STI is sexually transmitted it must have been acquired elsewhere [1], necessitating the involvement of one or more additional partners in a serial or parallel manner. Hypothetically, if two individuals had only one sexual partner during their lifetime, those two persons could hardly ever be affected by an STI. Even if the unlikely event of a non-sexual transmission of an STI were considered, it could not be further spread beyond those two individuals [1]. If the transmission efficiency of an infectious agent is poor, requiring a rather large number of sexual contacts to cause transmission, the dynamics of an epidemiological spread provides information on underlying sexual activity. For poorly transmittable infections, the average period of sexual activity before transmission occurs will be longer or the frequency of sexual contacts will be higher than for more easily transmittable infections.

The objective of this proof-of-principle modeling study was to provide a retrospective estimation of sexual activity among heterosexuals based on reported HIV incidence and prevalence in Germany. The intention behind this was to provide a simple and minimalistic approach to estimate sexual activity based on HIV incidence and prevalence. This study was independent of estimates provided by surveys, which may not be feasible in many situations according to social, cultural, and religious contexts. For modeling, we used a survey from Haversath et al. [2], which provided representative data on the sexual behavior of Germans and the official reported HIV incidence and prevalence [21], as well as published articles on the risk of HIV infection depending on sexual activity and the factors discussed above [22,23]. The basic hypothesis was the assumption that there is a correlation between the frequency of sexual activity and the likelihood of transmission of a poorly transmittable STI such as HIV infection, so that transmission of poorly transmittable STIs is an indirect indicator of sexual activity in a population.

## 2. Materials and Methods

Although modeling was aimed at a retrospective estimation of sexual contacts based on reported HIV incidence and prevalence, the inverse path from the number of sexual contacts to incidence and prevalence was pursued in a first step. This step was based on the estimation of HIV incidence and prevalence in Germany [21] and a representative survey on sexual behavior in Germany [2], which data were freely available for use. All technical details of the survey, including the details of data acquisition and ethical clearance, were available in the openly accessible source publication [2] and therefore will not be repeated here in detail.

### 2.1. Baseline Characteristics

According to previous works [22,23,24], we assumed that the incidence of sexually transmitted HIV infection depends upon the following:

— The HIV prevalence in a sexually active population
— The likelihood of transmission per number of sexual acts
— The number of risky sexual contacts (i.e., sexual contacts with someone with a confirmed or probable/possible STI)
— The proportion of those successfully treated who can no longer transmit the STI or who can only transmit it with a significantly lower probability

For HIV infections, transmission risk drops to an extremely low probability in the case of successful therapy, both in real life [25] and in statistical modeling [24]. If all other factors are known (prevalence, incidence, and the proportion of successfully treated individuals from the reported data, as well as transmission probability derived from the literature), this information can be used to estimate the number of risk contacts. Note that incidences attributable to sexual transmission have to be applied, as HIV can also be transmitted through other routes.

### 2.2. Risk of Infection According to the Number of Sexual Contacts and HIV Status of Each Partner

The following calculation of incidence is based on a survey by Haversath et al. [2] on sexual behavior in Germany.

The risk of transmission after a single extradyadic sexual contact is Bernoulli-distributed with an expected value p. It follows that the risk of transmission within n sexual contacts is binomially distributed and

(1) $$P\left(I\geq 1|C=n\right)=1-\left(\begin{array}{c} n\\ 0 \end{array}\right)p^{0}{\left(1-p\right)}^{n}=1-{\left(1-p\right)}^{n}$$

where I denotes the number of infectious contacts and C denotes the number of sexual contacts with infected partners.

Because Equation (1) holds only for an individual exposed to an infected sexual partner, the status of infection has to be taken into account. Therefore, for an individual’s risk of infection:

(2) $${\mathrm{Risk}}_{\mathrm{Infection}}=\overset{l}{\underset{k=1}{\sum }}{\mathrm{Status}}_{\mathrm{HIV}}\left(1-\overset{l}{\underset{k=1}{\prod }}{\left(1-p\right)}^{n_{k}}\right)$$

where l denotes the number of sexual partners, n_k_ the number of sexual contacts with partner k, and Status_HIV_ the HIV-status of partner k.

### 2.3. The Risk of Infection According to the Number of Sexual Contacts and the HIV Prevalence as Likelihood of a Positive HIV-Status of Each Partner

In the absence of information regarding the individual HIV status of each sexual partner and the number of sexual contacts per partner, as well as someone’s overall number of sexual contacts, the model given above can be adapted as follows.

Instead of individual HIV-status, we used the prevalence rate representing probability of a positive HIV status of a sexual partner. The following further simplifying assumptions were made:

The number of sexual contacts in the previous year was the same for all years during a sexually active life.

The number of sexual contacts was expected to be equally distributed over all sexual partners during the sexually active life.

It follows that the individual risk of infection over n contacts with l sexual partners and n/l sexual contacts per sexual partner is given by:

(3) $${\mathrm{Risk}}_{\mathrm{Infection}}=l*\mathrm{Prevalence}\;\mathrm{rate}\left(1-{\left(1-p\right)}^{n/l}\right)$$

### 2.4. The Number of Sexual Contacts During Life According to Given Incidence and Prevalence of HIV

For known mean numbers of sexual contacts during lifetime of a population and known HIV prevalence in this population, plus known risks of infection, incidence among the population at risk, denoted by Population_Risk_, is estimated by:

(4) $$\mathrm{Incidence}={\mathrm{Population}}_{\mathrm{Risk}}{*\mathrm{Risk}}_{\mathrm{Infection}}=l*\mathrm{Prevalence}\left(1-{\left(1-p\right)}^{n/l}\right)$$

Thus, it follows that

(5) $$1-\frac{\mathrm{Incidence}}{l*\mathrm{Prevalence}}={\left(1-p\right)}^{n/l}$$

For the expected number n of sexual contacts during lifetime in a population with given incidence and prevalence:

(6) $$n=l*\log_{1-p}(1-\frac{Incidence}{l*\mathrm{Prevalence}})$$

## 3. Results

In the survey on German sexual behavior by Haversath et al. [2], data were reported on the number of sexual contacts during the previous year, as well as during the month prior to questioning. The self-reported total number of sexual partners was also recorded (Table 1). The types of sexual contact (i.e., oral, vaginal, or anal) of each respondent were also documented in general but not differentiated for each individual sexual contact, because sexual preferences were reported but not every contact was categorized as such.

The HIV prevalence among heterosexual individuals in Germany can be assumed as a total of 30,000 [21]. Among these, 11,000 were infected by heterosexual contacts and about 19,000 otherwise (including infections acquired abroad, infections by men having sex with men, infections due to infusions and intravenous drug applications, etc.). The incidence of infections caused by heterosexual contacts is about 680 per year [21].

With the published incidence and prevalence rates [21], we estimated the expected number of sexual contacts during lifetime compared with the self-reported sexual activity in the survey [2].

We first provided an overall estimation for p, which is the expected value for the risk of transmission after a single extradyadic sexual contact. This was based on the transmission risk of HIV infection under treatment at the chronic stage from females to males and from males to females given by Hahn et al. [24], and weighted for the distribution of effectively treated individuals, as well as the distribution of sexually active German males and females (49.2% vs. 50.8%). For the situation under treatment, the transmission risk from females to males was given as 4.3 × 10^−5^, whereas for males to females it was 2.2 × 10^−5^. For the situation without treatment, it was 0.001 for transmission from females to males and 0.0005 for transmission from males to females.

Weighting these transmission risks for the distribution in Germany results in an overall transmission risk of 2.34 × 10^−4^ for unprotected sexual contacts and 4.68 × 10^−5^ for sexual contacts protected by a condom.

For the given prevalence of 30,000 and incidence of 680 per year, a median number of four sexual partners with risk of transmission p was found. Equation (6) gave an estimated median number of contacts during life among the population at risk of about *n* = 97 for exclusively unprotected sexual contacts and of about *n* = 486 for exclusively condom-protected contacts.

To combine these findings with those from the Haversath study [2], we estimated the overall number of sexual contacts for participants in this study. To do this, we further assumed an age of first sexual contact of 15.5 years, which is in accordance with an assessment by the German Federal Center for Health Education from 2015 [26]. Since there were survey respondents younger than 15.5 years, we defined per individual:

(7) $${\mathrm{Years}}_{\mathrm{sexual}\;\mathrm{activity}}=\max\left(1,\mathrm{age}\;\mathrm{in}\;\mathrm{years}-15.5\right)$$

With this assumption, an estimated number of overall sexual contacts per individual during life n is given as

(8) $$n={\mathrm{Years}}_{\mathrm{sexual}\;\mathrm{activity}}*\mathrm{number}\;\mathrm{of}\;\mathrm{sexual}\;\mathrm{contacts}\;\mathrm{during}\;\mathrm{last}\;\mathrm{year}$$

This results in an estimated median number of sexual contacts in the Haversath study [2] as 296, which corresponds to the estimated number of sexual contacts (97) without any HIV prevention and with exclusive condom use-based prevention (486) [21].

On the other hand, the estimated number of sexual contacts (296) [2] would lead to an estimated incidence of 560 for 100% adherence to condom use as a prevention strategy and 1789 for exclusively non-protected sexual contacts (Equation (4)).

Depending on the adherence to condom use as a protective measure, the HIV incidence of 680 due to heterosexual transmission [21] can be reproduced by the data of Haversath et al. [2] (Table 2) with an assumed condom use rate of 51.2%. Details of the calculations are provided in the Supplementary Materials S1 and S2.

## 4. Discussion

Given the incidence and prevalence of HIV infections as reported by the German National Health Authority, our model estimated that the median number of sexual contacts over the course of an individual’s lifetime ranges between 97 to 486, depending on condom use as a prevention strategy. This estimation corresponds to the estimated contacts (296) based on the self-reported sexual contacts reported in the Haversath study [2]. Our model confirmed the incidence of 680 sexually transmitted HIV infections per year among the German heterosexual population [21]. Thus, this study demonstrated the possibility of making a crude estimate of sexual activity based only on the incidence and prevalence of HIV infection.

Nevertheless, this study has a number of limitations. First of all, several assumptions about stable sexual activity over time were made because more detailed information was not available. The same applied to the distribution of sexual contacts per partner and the HIV status of each partner. For instance, the assumption of more or less stable sexual activity in relationships independent of external factors seems unrealistic, as a recent assessment made in the UK suggested declining frequency of sexual intercourse, especially among married or cohabiting individuals [5]. In addition, population-based modeling, as performed in the present study, do not reflect inter-individual characteristics that may affect the probability of adherence to preventive measures, such as condom use [27]. Finally, the entire sample from Haversath’s study [2] was assessed without direct comparison by gender. The Robert Koch Institute reports HIV prevalence but not heterosexual HIV incidence differentiated by gender, so a cross-gender modeling approach was chosen. However, the different infection risks of the sexes were still included in the calculation, because the risk of infection was weighted by gender risks and gender distribution.

Despite these uncertainties, we concluded that the number of contacts derived from the reported incidence confirmed the figures suggested by the questionnaire-based assessment [2]. As such, we can assume that HIV incidences correspond with the reported data on sexual contacts. However, some sources of uncertainty still remain, especially with regard to prevention approaches, which is relevant only for sexual contacts with HIV-infected partners. Although complete neglect of condom-based protection is not suggested by the data, we found that condoms were unlikely to be consistently used, since otherwise a lower HIV incidence would be expected based on the sexual activity level determined by the questionnaire-based assessment [2].

Because at least three individuals are required for STI transmission, as everyone has to acquire an infection from someone first in order to transmit it to another person, extradyadic sexual contacts or sequential changes of sexual partners are necessarily involved in STI transmission. For global modeling, however, it is neither relevant nor distinguishable for individual transmission events whether HIV transmission-associated sexual risk contacts have occurred within or outside of a traditional relationship involving only two people.

Approximately 51% of condom use calculated during this modeling exercise did not apply to all sexual contacts, but only to contacts “at risk”. This does not contradict even a considerable decline in condom acceptance. It can be postulated that even low condom acceptance is in line with the observed incidences as long as there is a degree of willingness to resume condom use in situations perceived as risky. Thus, realistic condom use of significantly less than 50% would fit well with the numbers used in the presented model.

It must be stressed that the model includes only individuals from the Haversath study with exclusively heterosexual sex partners according to self-disclosure. Although MSM (men having sex with men) accounts for the major share in sexual HIV transmission in Germany [21], this subpopulation was omitted in our model. Their number of approximately 19 in the Haversath study was too low to allow for reliable conclusions. In a calculation including the whole population, the MSM-subpopulation could not be ignored, but this was beyond the scope of this study.

## 5. Conclusions

The present study showed that a retrospective estimation of sexual activity in a population based on HIV incidence and prevalence was confirmed by data provided by Haversath’s representative survey [2]. This demonstrates that even minimal information such as incidence and prevalence can help estimate sexual activity if representative surveys are not feasible.

The results concluded in this study are likely to be useful for public health decision makers in situations where reliable data on sexual risk behavior are not otherwise available and the range of uncertainty is considered acceptable. The model may be particularly suitable for settings where severe reporting bias may be expected for legal or socio-cultural reasons, e.g., in societies where socially disapproved sexual behavior is legally proscribed and threatened with harsh judicial sentences.

What the study describes is a tool for researchers who are trying to roughly estimate sexual activity in regions where truthful self-reporting cannot be expected. This tool is based on association between the frequency of sexual activity and the likelihood of transmitting poorly transmittable STIs, such as HIV infections. Thus, the scope of this work is the model itself, while the reported numbers or proportions from Germany are merely used as a working example, not in themselves to be taken as having social or academic relevance in originating or promoting policies. The model was provided for the use of researchers whose research is intended to guide political decision makers but cannot be a criterion for political decision making in and of itself. Due to the limitations discussed above, the modeling approach delivers only a rough estimation of sexual activity within a population of interest. Therefore, it is not meant to be primarily used in socio-cultural settings where traditional questionnaire-based assessments are likely or more likely to provide realistic estimations. Even in such settings, however, the model can be applied to check the plausibility of questionnaire-based results.

## Figures and Tables

**Table 1:** Data on sexual behavior in Germany as derived from the study by Haversath et al. [2]. Only individuals from the Haversath study with exclusively heterosexual sex partners according to self-disclosure were included.

Categories	*n*	Mean (SD)	Median (q25; q75)
Age in years	1839	48.33 (18.31)	49 (34; 62)
Estimated time of sexual activity in years *	1839	32.87 (18.22)	33.50 (1; 76.5)
Number of male sexual partners	905	4.69 (7.05)	3 (1; 5)
Number of female sexual partners	884	8.95 (13.88)	5 (2; 10)
Number of sexual partners of other sex	1789	6.80 (11.17)	4 (1; 8)
Number of sexual contacts last year	1286	27.91 (41.37)	12 (0; 40)
Estimation of overall number of contacts during life **	1286	730.90 (1186.06)	296 (0; 1000)

* For the estimation of the time of sexual activity in years Equation (7) was used; ** for the estimation of overall number of contacts during life Equation (8) was used.

**Table 2:** Estimated mean numbers of sexual contacts and HIV incidence depending on use of condoms for prevention of transmission using Equations (4) and (6). Only individuals from the Haversath study [2] with exclusively heterosexual sex partners according to self-disclosure were included.

Categories	Only Unprotected Sexual Intercourse	Only Condom-Protected Sexual Intercourse
Transmission risk	2.34 × 10^−4^	4.68 × 10^−5^
Estimated mean number of contacts during life (with given HIV prevalence and incidence) *	97	486
Estimated HIV incidence (contacts per year-based estimated number of contacts during life)	2060	415

* Prevalence among heterosexuals 30,000; incidence of heterosexual contacts among heterosexuals 680.

## Supplementary Materials

The following are available online at https://www.mdpi.com/1660-4601/17/15/5504/s1. Supplementary material S1: The incidence according to given risk of infection, number of sexual partners, and number of sexual contacts during life, as well as prevalence of HIV. Supplementary material S2: The number of sexual contacts during life according to given incidence and prevalence of HIV.
