# Supplementary material for: Comparison of Self-Reported Sexual Activity Among Heterosexuals with Sexual Spread of Poorly Transmittable Agents: A Minimalistic Approach to Estimating Sexual Activity Based on HIV Incidence
Source: Int J Environ Res Public Health. 2020 Jul 30;17(15):5504. doi: 10.3390/ijerph17155504 (PMC7432220; doi:10.3390/ijerph17155504)
Supplement: Supplementary file 1 [file ijerph-17-05504-s001.pdf]

Supplementary Material

S1. The Incidence According to Given Risk of Infection, Number of Sexual Partners, and Number of Sexual Contacts During Life, as well as Prevalence of HIV

For known mean numbers of sexual contacts during one's lifetime and known HIV prevalence and known risks of infection, an estimation for incidence among the population at risk, denoted by $\text{Population}_{\text{Risk}}$, is given:

$$\text{Incidence} = \text{Population}_{\text{Risk}}\text{Risk}_{\text{Infection}} = l * \text{Prevalence}\left(1 - (1-p)^{n/l}\right) \quad (4)$$

HIV prevalence among heterosexuals is about 30,000 individuals. The median number of sexual partners is 4 (see Table 1). The median number of sexual contacts during the previous year was 12.

The estimated number of years with sexual activity, as adapted from the Harversath study [2], was defined in our model as:

$$\text{Years}_{\text{sexualactivity}} = \max\,(1, \text{age in years} - 15.5) \quad (7)$$

Based on this assumption, an estimated number of sexual contacts, *n*, per individual is:

$$n = \text{Years}_{\text{sexualactivity}} * \text{number of sexual contacts during last year} \quad (8)$$

This resulted in the median number, n, of sexual contacts to be 296 among the heterosexual study population.

S2. The Number of Sexual Contacts during Life According to Given Incidence and Prevalence of HIV

For the estimated number n of sexual contacts over the course of an individual's lifetime based on given incidence and prevalence, we have:

$$n = l * \log_{1-p}\left(1 - \frac{\text{Incidence}}{l * \text{Prevalence}}\right) \quad (6)$$

The HIV incidence per year was given as 680 individuals, whereas the prevalence was 30,000 individuals [21] and the median number l of sexual partners during life was 4.

The overall estimation for p, which is the expected value for the risk of transmission after a single extradyadic sexual contact, was based on the transmission risk under treatment and at chronic stage of transmission from females to males and from males to females [24]. These findings were weighted for distribution in effectively treated individuals (71.7%) and the distribution of German males and females. For the situation under treatment, the transmission risk from females to males was 4.3×10^{-5} and from males to females was 2.2×10^{-5}. For the situation without treatment, it was 0.001 for transmissions from females to males and 0.0005 for transmissions from males (49.2%) to females (50.8%).

For the overall p, we estimated:

$$p = 0.492(0.717 * 4.3 \times 10^{-5} + (1 - 0.717) * 0.001) + 0.508(0.717 * 2.2 \times 10^{-5} + (1 - 0.717) * 0.0005) = 2.34 \times 10^{-4}$$

Using (4), for exclusively non-protected contacts, we found:

$$97 = 4 * \log_{1-2.34\times10^{-4}}\left(1 - \frac{680}{4 * 30{,}000}\right)$$

For exclusively condom-protected contacts, the result was:

$$486 = 4 * \log_{1-4.68\times10^{-5}}\left(1 - \frac{680}{4 * 30{,}000}\right)$$

For the estimated median number of 296 contacts taken from the Harversath study [2], these results correspond to a condom use rate c of 0.512:

$$296 = 97(1 - c) + 486c = 97 + 389c$$
